# Unraveling the enigma: The emerging significance of pulmonary surfactant proteins in predicting, diagnosing, and managing COVID‐19

**DOI:** 10.1002/iid3.1302

**Published:** 2024-06-11

**Authors:** Mohammad Navid Bastani, Shahram Jalilian

**Affiliations:** ^1^ Department of Medical Virology, School of Medicine Ahvaz Jundishapur University of Medical Sciences Ahvaz Iran

**Keywords:** COVID‐19, pulmonary surfactant protein, SARS‐COV2, SP, surfactant proteins

## Abstract

**Background:**

Severe cases of COVID‐19 often lead to the development of acute respiratory syndrome, a critical condition believed to be caused by the harmful effects of SARS‐CoV‐2 on type II alveolar cells. These cells play a crucial role in producing pulmonary surfactants, which are essential for proper lung function. Specifically focusing on surfactant proteins, including Surfactant protein A (SP‐A), Surfactant protein B, Surfactant protein C, and Surfactant protein D (SP‐D), changes in the levels of pulmonary surfactants may be a significant factor in the pathological changes seen in COVID‐19 infection.

**Objective:**

This study aims to gain insights into surfactants, particularly their impacts and changes during COVID‐19 infection, through a comprehensive review of current literature. The study focuses on the function of surfactants as prognostic markers, diagnostic factors, and essential components in the management and treatment of COVID‐19.

**Finding:**

In general, pulmonary surfactants serve to reduce the surface tension at the gas–liquid interface, thereby significantly contributing to the regulation of respiratory mechanics. Additionally, these surfactants play a crucial role in the innate immune system within the pulmonary microenvironment. Within the spectrum of COVID‐19 infections, a compelling association is observed, characterized by elevated levels of SP‐D and SP‐A across a range of manifestations from mild to severe pneumonia. The sudden decline in respiratory function observed in COVID‐19 patients may be attributed to the decreased synthesis of surfactants by type II alveolar cells.

**Conclusion:**

Collectin proteins such as SP‐A and SP‐D show promise as biomarkers, offering potential avenues for predicting and monitoring pulmonary alveolar injury in the context of COVID‐19. This clarification enhances our understanding of the molecular complexities contributing to respiratory complications in severe COVID‐19 cases, providing a foundation for targeted therapeutic approaches using surfactants and refined clinical management strategies.

## Highlights

- SARS‐CoV‐2 affects alveolar type II cells, which limits the synthesis and secretion of pulmonary surfactant into the alveolar space, ultimately leading to lung failure.
- Surfactant proteins A and D, as members of the lectin‐C family, play a prominent role in COVID‐19 infection. SP‐D exhibits a higher affinity for binding the spike protein of SARS‐CoV‐2 compared to SP‐A.
- In COVID‐19 infection, the levels of SP‐D and SP‐A were significantly higher in cases of mild to moderate pneumonia than in severe or critical cases.

## Graphical abstract

An increase in SP‐A and SP‐D amount has been seen in the moderate COVID‐19, but in the severe stages of the disease due to the destruction of type 2 alveolar cells, which are the main producers of surfactants, these amounts will be reduced.

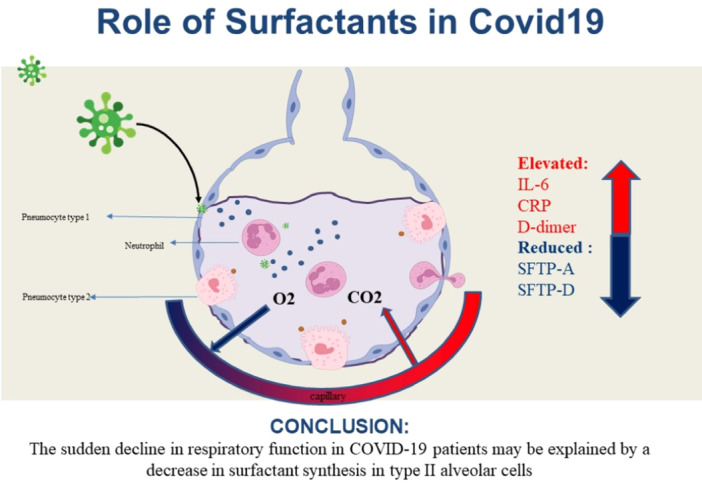

## 1 INTRODUCTION

### 1.1 COVID‐19

In December 2019, an outbreak of pneumonia with an unknown cause was reported in Wuhan, a city in Hubei province, China. The outbreak was linked to the Huanan Seafood Wholesale Market. The World Health Organization (WHO) identified the causative agent of the outbreak as the severe acute respiratory syndrome coronavirus‐2 (SARS‐CoV‐2), which causes coronavirus disease‐2019 (COVID‐19). As a result of the virus spreading to at least 200 countries, the WHO declared COVID‐19 a pandemic. As of now, there have been approximately 635 million confirmed cases and 6.5 million deaths worldwide, with daily increases in the number of cases.^1^, ^2^

Coronaviruses (CoVs) are enveloped icosahedral symmetric particles with a diameter ranging from 80 to 220 nm. They have a nonsegmented, single‐stranded, positive‐sense RNA genome that varies in size from 26 to 32 kb. CoVs belong to the Nidovirales order and are a significant group of viruses. The name “coronavirus” is derived from the spike projections on the virus envelope, which give it a crown‐like appearance under electron microscopy.^1^, ^2^

Coronavirus genomes range in length from 26 to 32 kb and contain a varying number of open reading frames (ORFs). It has been discovered that the SARS‐CoV‐2 genome contains 14 ORFs that encode 27 proteins. The spike surface glycoprotein is necessary for attaching to receptors on the host cell, defining host tropism and transmission capacity, facilitating receptor binding, and mediating membrane fusion. The spike protein of CoVs is functionally separated into two domains: the S1 domain, which is necessary for receptor binding, and the S2 domain, which is responsible for cell membrane fusion. The SARS‐CoV‐2 genome contains eight accessory proteins (3a, 3b, p6, 7a, 7b, 8b, 9b, and orf14) and four major structural proteins.^3^

### 1.2 Pathophysiology of surfactants in SARS‐CoV‐2 infection

The pathophysiology of SARS‐CoV‐2 infection in humans manifests as mild symptoms to acute respiratory failure (ARF). The virus replicates and travels through the respiratory tract by attaching to epithelial cells and penetrating alveolar epithelial cells (AECs) in the lungs. The rapid replication of SARS‐CoV‐2 in the alveoli can trigger a robust immunological response, leading to cytokine storm syndrome and acute respiratory distress syndrome (ARDS), which is the main cause of death in COVID‐19 patients.^4^ Most histopathological abnormalities in COVID‐19 patients are found in the lungs, including bilateral diffused alveolar destruction, hyaline membrane development, pneumocyte desquamation, and fibrin deposits. Exudative inflammation was also observed in some cases. Immunohistochemistry revealed SARS‐CoV‐2 antigen in the upper airway, bronchiolar epithelium, submucosal gland epithelium, type I and type II pneumocytes, alveolar macrophages, and hyaline membranes in the lungs.^5^

SARS‐CoV‐2 affects alveolar type II cells, which is responsible for pulmonary surfactant synthesis and secretion into the alveolar space. The virus's spike protein's receptor‐binding domain is associated with the angiotensin‐converting enzyme receptor 2 (ACE2), which is located on alveolar epithelial type II cells.^6^ SARS‐CoV‐2 interacts with the ACE2 receptor, disrupting type II alveolar cells. The function of the lungs depends on the performance of type II epithelial cells, which produce and release alveolar surfactant, reducing the collapsing forces caused by the liquid–air interface in the alveolar epithelium. When the amount of pulmonary surfactant synthesized and secreted into the alveolar space is reduced, pulmonary atelectasis and ARDS can occur. Lung surfactant failure in COVID‐19 patients can disrupt the air–liquid interface, leading to a decrease in blood oxygen levels, delayed restoration, edema, lung fibrosis, and systematic respiratory collapse.^7^

**Figure 1:**
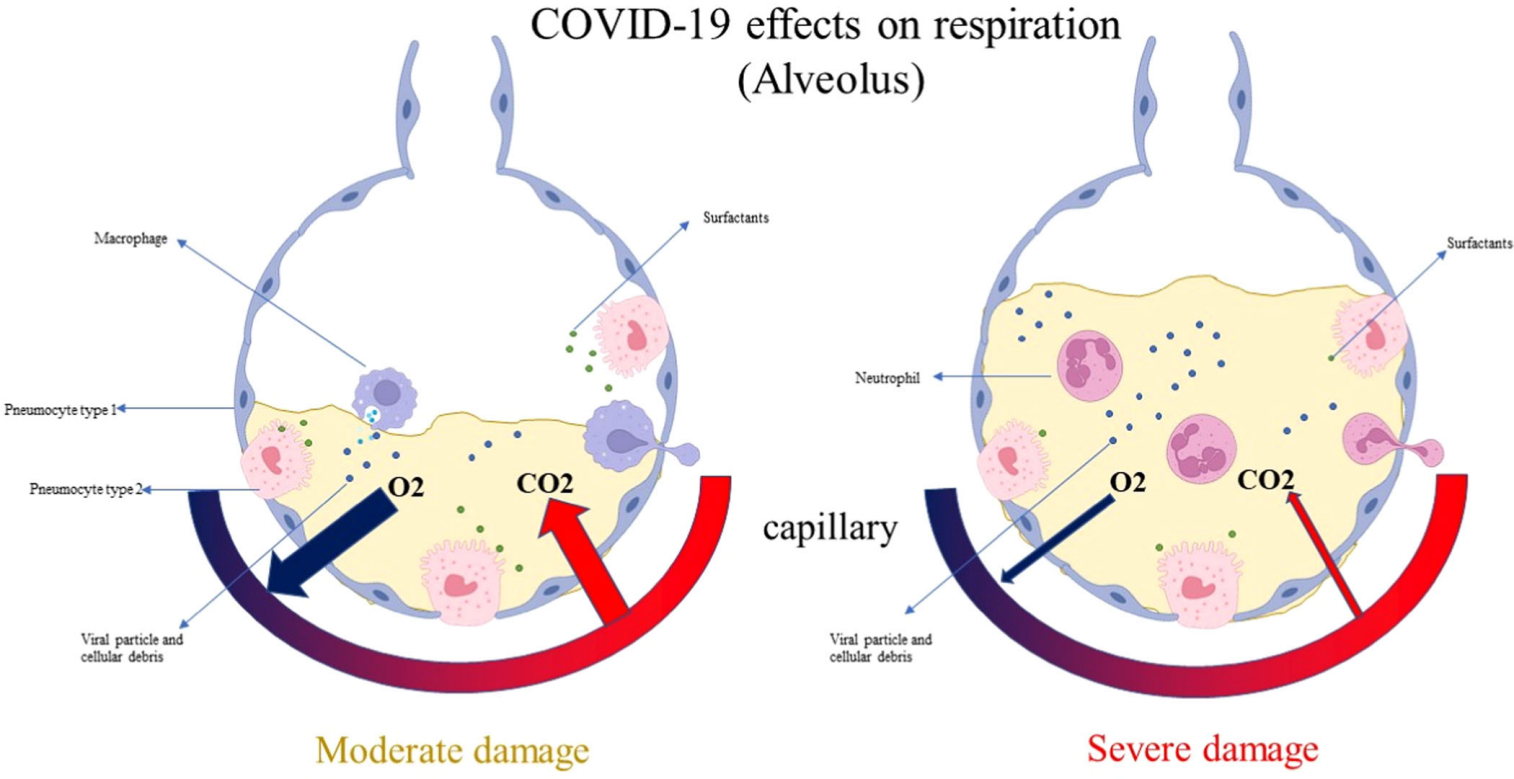
Schematic figure of SARS‐CoV‐2 infection in respiratory in two manners. Moderate infection: accumulation of fluid in the alveolar and reduced gas exchange. Severe damage: submersion of alveolars and very limited gas exchange.

Most COVID‐19 patients admitted to intensive care units have clinical manifestations of ARDS. According to emerging research on respiratory dynamics, the clinical symptoms of ARDS in COVID‐19 patients match those of ARDS caused by surfactant insufficiency^8^ (Figure 1).

## 2 RESPIRATORY SURFACTANTS

The alveolar region of the pulmonary system plays a crucial role in the exchange of oxygen and carbon dioxide for respiration. Surfactant is composed of about 90% lipids, primarily phospholipids like dipalmitoyl phosphatidylcholine which plays a pivotal role as the most efficacious constituent in reducing surface tension and 10% proteins, including Surfactant protein A (SP‐A), Surfactant protein B (SP‐B), Surfactant protein C (SP‐C), and Surfactant protein D (SP‐D). SP‐A and SP‐D are both soluble in water, but SP‐B and SP‐C are highly hydrophobic.^9^ Each of the four surfactant proteins (SPs) has unique structures and functions and is highly expressed in type II cells. Surfactant components are tightly regulated to maintain accurate levels throughout life, with type II cells recycling the surfactant elements or alveolar macrophages catabolizing them. Surfactant lipids and proteins are predominantly produced by type II cells, with massive intracellular organelles called lamellar bodies containing surfactant lipids like phosphatidylcholine and phosphatidylglycerol. These two hydrophobic low‐molecular‐weight proteins are secreted alongside the SPs. The biosynthesis of SPs and lipids is coordinated with recycling/degradation processes to maintain intracellular and alveolar surfactant pool size.^10^

The secretion of surfactant from alveolar type II cells is a regulated process that ensures surfactant is available in the alveoli when needed. This process is primarily controlled through the exocytosis of lamellar bodies, the organelles where surfactant components are stored. The regulation of surfactant secretion involves various signaling pathways and molecules, including calcium and ATP.^11^ One key regulator of surfactant secretion is the intracellular calcium concentration. An increase in intracellular calcium triggers the fusion of lamellar bodies with the plasma membrane, leading to the exocytosis of surfactant. This calcium‐mediated secretion can be stimulated by mechanical stretching of the lungs, which mimics the physiological process of breathing.^12^

In addition, Ig‐Hepta/GPR116 controls pulmonary surfactant levels by monitoring SP‐D. Ig‐Hepta/GPR116 is a G protein‐coupled receptor (GPCR) that contains immunoglobulin (Ig)‐like repeats known as adhesion GPCRs. It is predominantly expressed in lung alveolar type II epithelial cells and is involved in monitoring lung surfactant levels. The physiological role of Ig‐Hepta/GPR116 is unknown; however, it has been linked to various activities including activation of alveolar macrophages.^13^

SP‐B and SP‐C are believed to modify the packing and spreading of lipids, decreasing surface tension, lowering lipid activity, and regulating the lipid layers during respiration. SP‐A and SP‐D, on the other hand, are larger, more numerous oligomeric proteins produced and released by type II cells. SP‐D and SP‐A are members of the collectin family of C‐type mammalian lectins with unique collagen‐like and globular carbohydrate‐binding domains. SP‐A is essential for tubular myelin development and performing various roles in lung host‐defense processes. It binds to lipopolysaccharides and different microbial pathogens, facilitating their clearance from the lung. Unlike SP‐B and SP‐C, SP‐A does not play a critical role in surface functions, metabolism, or pulmonary surfactant under normal circumstances. SP‐D, on the other hand, impacts the conformations of pulmonary surfactants and plays a significant role in regulating pulmonary surfactant pool sizes and reuptake.^14^, ^15^ It is also necessary for controlling pulmonary inflammation and host defense against viral, fungal, and bacterial infections. Surfactant lipids and proteins (Table 1) help reduce surface tension in the alveolus, which is vital for ventilation and modulates several features of the lung's innate immunity against various pulmonary infections.^12^

**Table 1:** Characterization of pulmonary surfactant protein.

Protein	Characterization	Chromosomal location	General functions
SP‐A1 SP‐A2	Hydrophilic, collagen‐containing C‐type lectin, part of the collectin family, most abundant surfactant protein	10q22.3‐ Exon 12 10q22	Reduces surface tension, modulates pulmonary host defense, stimulates chemotaxis and phagocytosis of alveolar macrophages, binds pathogens, regulates immune cell function, serves as an opsonin for microbial clearance, and modulates inflammatory responses.^16^
SP‐B	Hydrophobic, essential for air‐breathing, conserved among mammals	2p11.2	Critical for lowering surface tension, essential for the structural integrity and function of surfactant, facilitates the spread of surfactant phospholipids.^17^
SP‐C	Hydrophobic, unique structure, localized in the lung	8p21.3	Contributes to the surface tension‐lowering properties of surfactant, important for the evolutionary origin of surfactant.^18^
SP‐D	Hydrophilic, collagen‐containing C‐type lectin, part of the collectin family	10q22.3‐ Exon 9	Participates in innate immune defense, binds and clears various pathogens, modulates immune responses, and regulates inflammatory processes.^19^

### 2.1 Surfactant protein‐A1 (SP‐A1) and surfactant protein‐A2 (SP‐A2)

SP‐A is encoded by two genes in humans and primates (unlike rodents), SP‐A1 and SP‐A2. The two genes are separate, although they have a significant degree of sequence similarity. The SP‐A locus is located on chromosome 10q22.3 and contains two functional genes, SP‐A1 and SP‐A2, that are transcriptionally oriented in different directions.^20^

SP‐A is the most prevalent of the four types of proteins and a member of the alveolar superfamily, which is known to participate in innate immune defense. SP‐A1 appears to be involved in pulmonary surfactant rearrangement, as well as effective phospholipid adsorption at the air–liquid interface. As a result, adequate quantities of SP‐A1 may be required to ensure mechanical stability and breathing efficiency.^21^ SP‐A2 appears to have stronger activity in terms of its capacity to accelerate phagocytosis by alveolar macrophages, increase cytokine production by a macrophage‐like cell line, and limit surfactant synthesis by alveolar epithelial type II cells.^22^

SP‐A not only acts as a structural protein in the lungs to relieve surface tension but also acts as an innate immune protein in the lungs. SP‐A enhances pathogen clearance via opsonization and phagocytosis. The collectin‐recognizing domain binds to the pathogen surface to achieve SP‐A pathogen opsonization. The interaction between the collectin and the immune cell promotes pathogen phagocytosis. SP‐A improves opsonic and nonopsonic phagocytosis in macrophages through binding to the SP‐A receptor.^23^ In viral infections like respiratory syncytial virus (RSV) infection, SP‐A can bind and destroy the virus. This collectin has an important role in safeguarding the immunologically naive human newborn against RSV infection.^24^, ^25^

SP‐A also controls the inflammatory response by reducing the expression of inflammatory cytokines. SP‐A can reduce tumor necrosis factor (TNF)‐α secretion and nitric oxide production in triggered macrophages, balancing the inflammatory response and preventing immune‐mediated pulmonary pathology, considered the main reason for COVID‐19 respiratory distress.^26^ Equally, SP‐A interacts with toll‐like receptors (TLRs) 2 and 4 to modulate inflammatory responses triggered by viral exposures. TLR activation via SP‐A regulates the generation of cytokines and inflammatory mediators such as TNF and interleukin (IL)‐1.^27^ SP‐A also inhibits the synthesis of IL‐8 in eosinophils. IL‐8, which is generated by macrophages and epithelial cells, causes acute lung injury (ALI) via SP‐A.^28^ Silencing IL‐8 enhances SP‐A and Bcl‐2 expression while decreasing other apoptosis‐related proteins and immune cell regulators, resulting in reduced apoptosis and enhanced cell survival. Silencing SP‐A, on the other hand, increases the expression of IL‐8, apoptotic proteins, and immune cell regulators and lowers Bcl‐2. Moreover, IL‐8 interacts with SP‐A to boost inflammatory cell response, finally leading to ALI. These findings imply that IL‐8 may aggravate damage to AECs by suppressing SP‐A production, thereby promoting lung inflammation.^29^ It should be mentioned, SP‐A has been demonstrated to reduce surfactant phospholipase A2 action, and the hydrolysis of surfactant phospholipids, which protects lung integrity, particularly in ARDS in an investigation SP‐A knockout mice had considerably greater levels of surfactant phospholipid hydrolysis, indicating that SP‐A inhibits this process and may play a role in ARDS.^30^

The interaction between SP‐A and TLR4 is crucial for host defense. The host defense activity of the SPA4 peptide is generated from the TLR4‐interacting region of SP‐A, which has antibacterial activity. SPA4 peptide therapy minimizes bacterial burden, inflammatory cytokine and chemokine development, lactate levels, alveolar edema, and tissue injury.^31^ A study compared its ability to reduce bacterial infection and demonstrated that a recombinant truncated human SP‐A peptide enhances influenza A virus (IAV) replication in lung epithelial cell line A549 when confronted with pH1N1 and H3N2 IAV subtypes, as evidenced by an increased expression of M1 protein, an important determinant of viral replication. Human full‐length native SP‐A, on the other hand, downregulates the M1 expression in A549 cells challenged with IAV subtypes, implying that the entire SP‐A molecule is essential for IAV protection.^32^ Furthermore, in RSV infection, studies demonstrate that employing SP‐A proves highly efficient in preventing subsequent infections and decreasing inflammatory cells of bronchoalveolar lavage in mice with knockout surfactant genes.^24^

SP‐A has been extensively validated as a serum biomarker for diagnosing lung disorders. Furthermore, it has been linked to the pathophysiology of idiopathic pulmonary fibrosis (IPF) and holds promise as a biomarker for assessing the efficacy of antifibrotic medication therapy in IPF patients.^33^ SP‐A is being explored as a potential biomarker for IPF, a chronic and progressive lung disease with poor outcomes. Alongside proteins like SP‐D, SP‐A is implicated in IPF's key aspect, epithelial cell injury. Elevated serum SP‐A levels have been linked to IPF, distinguishing it from other lung diseases and healthy states. Higher SP‐A levels are associated with increased mortality risk in IPF patients, hinting at its prognostic potential. Yet, validating SP‐A's role in IPF diagnosis, prognosis, and treatment response requires further large‐scale studies.^34^, ^35^ SP‐A has the potential to be utilized as a biomarker for prognosticating the prognosis of chronic obstructive pulmonary disease (COPD). Diminished levels of SP‐A, which are associated with compromised immunity, could heighten the vulnerability to exacerbations of COPD.^16^ Reduced levels of SP‐A, as seen in chronic lung ailments, could elevate the likelihood of viral infections and disease flare‐ups. This indicates that people with diminished levels of these proteins might face higher susceptibility to severe COVID‐19.^36^

It's worth mentioning the correlation of single nucleotide polymorphisms (SNPs) in pulmonary SP genes with ARF and its morbidity and pulmonary dysfunction at discharge (PDAD) reveal the highlighted roles of SP‐A1 and SP‐A2 in pathogenesis development. The SP‐A1 SNPs with the highest number of interactions (rs1136450, rs1136451) are found in the SP‐A1 collagen‐like domain. The rs1136451 mutation at coding region 62 is silent, whereas the rs1136450 mutation at coding region 50 alters the amino acid from leucine to valine, leading to increased PDAD.^37^ Another recently reported SNP is a heterozygous single nucleotide variation c.532G>A. This variant encodes the alteration p.[Val178Met], which is found in the carbohydrate recognition region of SP‐A and co‐segregates with the genes responsible for idiopathic interstitial pneumonia.^38^

Most intragenic and intergenic interactions linked with higher ARF risk (32%) involve SP‐A2 SNPs. The SP‐A2 SNPs (rs1059046 and rs1965708) had the most interactions in ARF pathogenesis. SP‐A2 rs1059046 and rs1965708 have been linked to severe RSV illness with high viral load resulting in ARF.^39^, ^40^

### 2.2 SP‐B

SP‐B belongs to the Saponin‐like protein [SAPLIN] family, which plays various roles such as interacting with lipids and exhibiting antimicrobial properties. SP‐B is a homodimer consisting of two 79‐amino acid polypeptide chains linked together by disulfide bonds. It is encoded by a single gene on human chromosome 2. The SP‐B precursor can produce two proteins, SP‐BM and SP‐BN, which are associated with surface tension reduction and innate host defense, respectively. Additionally, SP‐B is crucial for the production of tubular myelin and enhances the rate of phospholipid adsorption from an aqueous subphase to an air–liquid interface.^41^ Moreover, SP‐B induces pore formation in planar lipid membranes, which could potentially disrupt viral envelopes or membranes, although this is not a direct antiviral action.^42^

The first genetic abnormality associated with surfactant failure is SP‐B insufficiency. This autosomal recessive disease is caused by loss‐of‐function mutations on both SP‐B alleles. The lung disease progresses and is unresponsive to medical treatment, typically resulting in respiratory failure and death within 3 months of birth. Lung transplantation has been performed in neonates with SP‐B defects.^43^ ARDS patients experience severe respiratory failure or ARDS when SP‐B levels fall below 60% of normal. Multiple SP‐B polymorphisms and variations, including the SNP [rs1130866], have been identified as significant variation associated with various lung injuries, including pneumonia‐induced ARDS, pneumonia, and neonatal respiratory distress syndrome.^44^, ^45^

SP‐B‐derived peptides (SP‐BN) are fragments derived from SP‐B, these small homodimeric peptides have attracted attention due to their antiviral properties and potential therapeutic applications. They function through various mechanisms, including physical inhibition, hindering viral entry, enzymatic degradation of viral infected cells, and disruption of viral replication processes. Additionally, SP‐BN has shown efficacy in reducing lung inflammation by regulating inflammatory markers.^46^ When combined with other materials or technologies, such as nanostructured aluminum surfaces, SP‐BN can augment antiviral effects, especially in neutralizing viruses like SARS‐CoV‐2.^47^ Furthermore, Studies have demonstrated that synthetic lung surfactant peptide analogs, like Super Mini‐B and B‐YL, possess affinities to the recombinant human ACE2 receptor comparable to those of the viral spike protein. This implies that these synthetic peptides may function as competitive inhibitors, potentially obstructing the virus from attaching to the ACE2 receptor, thus either preventing infection or mitigating its severity.^48^

### 2.3 SP‐C

SP‐C is the smallest and most hydrophobic component of lung surfactant, weighing in at 4.2 kDa. It regulates inflammation as well as having biophysical activities. Loss of SP‐C is associated with increased inflammation and delayed restoration in both human and mouse models. Despite this, the primary mechanisms of SP‐C‐regulated inflammation in response to injury remain unknown.^49^ SP‐C is predominantly expressed in alveolar type II cells, which are facultative stem cells. When mature, SP‐C is highly hydrophobic and helps reduce surface tension during breathing. In addition to regulating the biophysical properties of alveolar surfactant phospholipids in the alveolus, the importance of SP‐C's structure, function, and biosynthesis has been highlighted by the heterozygous expression of more than 14 different mutant pro‐SP‐C types associated with the progression of diffuse parenchymal lung disease in both adult and pediatric patients.^50^, ^51^ Mutations in SP‐C can cause significant lung disease in older children and adults, and respiratory disease due to SP‐C mutations is inherited in an autosomal dominant manner or can be a consequence of de novo mutations causing an apparent sporadic disorder. A mutation on one allele can cause disease, and SP‐C mutations are a significant cause of diffuse lung disease, with the I73T polymorphism being the most common SP‐C mutation related to the disease.^52^ The age at which the disease manifests itself and the severity of the disease are highly unpredictable. Some people are asymptomatic until their fifth or sixth decade when they develop pulmonary fibrosis. According to a recent Dutch study, SP‐C mutations account for 25% of familial pulmonary fibrosis kindreds.^53^, ^54^

SP‐C modulates the permeability and dynamics of phospholipid membranes and facilitate cholesterol dynamics, which could indirectly affect viral entry or fusion with host cells.^55^, ^56^

Hence, recent investigation has revealed that SARS‐CoV‐2 infection generates autoantibodies against different cytokines and autoimmune disease‐related proteins in individuals with severe conditions,^57^ Individuals with severe COVID‐19 have IgA autoantibodies against lung SP‐B and SP‐C. These autoantibodies disrupt the function of lung surfactants, perhaps resulting in alveolar collapse and decreased oxygenation and triggering ARDS.^58^

### 2.4 SP‐D

SP‐D is encoded by a single gene on human chromosome 10, which is located adjacent to the SP‐A gene. While SP‐D was initially found in the lungs and is mainly produced by type II and other nonciliated bronchiolar respiratory epithelial cells, SP‐D mRNA and protein have also been found in various nonpulmonary tissues, including the intestine, bladder, and parotid glands. Extra‐alveolar levels of SP‐D are several‐fold lower than those detected in the lungs.^59^ SP‐D is a pattern‐recognition molecule in the collectin family. As an essential element of innate immunity, this soluble protein is classified as a pattern recognition receptor (PRR). PRRs are responsible for recognizing pathogen‐associated molecular patterns, including viral glycoproteins. The activity of human SP‐D promotes interfaces with microbial ligands that are glycosylated with a variety of saccharides. SP‐D has the ability to bind various inhaled microorganisms. It can also bind saccharides, lipids, and nucleic acids with a wide range of specificity, allowing it to initiate phagocytosis. The main roles of SP‐D include promoting the aggregation and rapid phagocytosis of microorganisms, apoptotic cells, and allergens. These functions are essential for the maintenance of healthy lungs that are both infection‐free and efficient in gas exchange.^60^

SP‐D is known to play a vital role in the prevention of Influenza A infection, inducing viral aggregation by binding to Hemagglutinin. the aggregation of viral and nonviral pathogens is a powerful strategy through which SP‐D assists in first‐line host defense in the lungs.^61^, ^62^

SP‐D has been proposed as a valuable biomarker for acute lung injuries and ARDS, a protective factor in various causes of ALI. Elevated levels of SP‐D in the blood are related to lung function during acute lung damage such as COVID‐19 infection.^63^ Both circulating SP‐D levels and SP‐D genetic variants are associated with the onset, progression, and exacerbation of various pulmonary illnesses, including ALI, IPF, ARDS, and COPD. For instance, the SNP Met11Thr (rs721917) in the coding sequence of the SP‐D gene has a significant effect on asthma and COPD. The SP‐D Met11Thr polymorphism also explains the susceptibility and severity of RSV bronchiolitis during infancy. These polymorphisms may have functional consequences as they change the amino acid structure of the mature protein. The hydrophobic characteristics of the two alternative peptides, Met and Thr, at position 11 in the mature SP‐D molecule differ significantly. Amino acid 11 is close to the conserved amino acids Cys15 and Cys20, which are important in the interchain disulfide bonding of the SP‐D molecule's trimeric subunits. These amino‐terminal disulfide bonds are essential for SP‐D's structural stabilization and its antiviral activities. Increasing the multimerization of SP‐D improves its antiviral efficacy. In addition, three SP‐D SNPs, rs2245121, rs911887, and rs6413520, were also linked to COPD susceptibility.^64^, ^65^, ^66^

The association of serum SP‐D with the mortality rate in pulmonary disorders, including COPD, underscores the importance of disease‐induced levels of SP‐D in prognosis.^67^, ^68^

## 3 PULMONARY SPs AND COVID‐19

In the COVID‐19 pandemic, it is estimated to have resulted in 683 million cases with a 3% fatality rate. The most common symptoms are anosmia/dysgeusia, conjunctivitis, diarrhea, and low‐grade fever. However, severe symptoms such as interstitial pneumonia, myocarditis, acute renal damage, ARDS, multiple organ failure, and death can also occur.^5^ The entry of SARS‐CoV‐2 into cells requires angiotensin‐converting enzyme receptors that are abundant in respiratory epithelial cells, including type II AECs. SPs are used to facilitate gaseous exchange at the air–liquid interface in the alveoli and also have the ability to control the actions of innate immune cells in the lung for pathogens eradication. They also inhibit inflammatory responses and facilitating removal of apoptotic cells following viral infection.^69^ Therefore, it became clear that viral regulation of surfactant metabolism could be a strong lever for pathological changes in the respiratory system.^70^ SARS‐CoV‐2 can alter all surfactant levels, including reduction in the level of SP‐B.^71^ In a recent investigation, it has been elucidated that during the course of COVID‐19 infection, soluble IgA autoantibodies targeting B and C surfactants manifest Autoantibody IgA disrupts the capacity of pulmonary surfactant to reduce surface tension. Consequently, this may result in compromised stabilization of pulmonary alveoli, thereby contributing to alveolar collapse and inadequate oxygen exchange resulting in a diminution of these surfactants and subsequent impairment of their functionality.^58^ This occurrence may represent a pivotal determinant in the pathogenesis of COVID‐19 and its consequential respiratory manifestations.^58^ Another study demonstrated that the destruction and reduction of AECs and subsequent reduction of pulmonary surfactants were associated with a higher risk of IPF.^72^ Furthermore, SARS‐CoV‐2 significantly downregulated the extracellular matrix protein annexin A2, which is responsible for mediating the secretion of lamellar bodies containing surfactant. SP‐A and SP‐D, as members of the lectin‐C family, seem to be playing a more significant role in COVID‐19 infection than any other surfactant. Since the entry into the lungs, SARS‐CoV‐2 is postulated to damage type II alveolar cells, the region that produces lung surfactants, resulting in the downregulation of pulmonary surfactants. Direct interaction of SP‐A and SP‐D with certain viruses leads to viral neutralization and improved phagocytosis more frequently. Both SP‐A and SP‐D limit the attachment activity of the IAV's hemagglutinin. However, SP‐D goes a step further by also inhibiting neuraminidase activity. As a result, SP‐D is more effective in reducing the infectivity of the IAV. It promotes significant aggregation of IAV particles, inhibits the functions of both hemagglutinin and neuraminidase and neutralizes viral particles.^73^, ^74^, ^75^

KL‐6, referred to Krebs von den Lungen 6, is a high weight mucin discovered in humans. It has been observed to be higher than normal in individuals with COVID‐19, especially in those with moderate to severe symptoms. Compared to the increase in the lung‐specific biomarker KL‐6,^76^ the increase in SP‐A and SP‐D initiated at an earlier stage in pneumonia.^36^ The scores derived from the analysis of chest CT scans exhibited a noteworthy and positive association with the levels of serum SP‐A and SP‐D.^77^ As pneumonia advanced from a nonsevere state to a severe one, there was a substantial rise in the levels of serum SP‐A and SP‐D, which was closely accompanied by clinical observations and CT scans. Conversely, the levels of SP‐A were significantly higher in cases of nonsevere pneumonia as compared to those observed in individuals with no medical abnormalities. Nonsevere instances of COVID‐19 encompassed patients who displayed normal chest X‐rays and exhibited mild pneumonia that was exclusively detectable through chest CT scans. Clinicians have the potential to identify the existence of slight pneumonia when serum SP‐A levels surpass predetermined thresholds. It has been demonstrated that pulmonary alveolar injury can be monitored using collectin proteins as biomarkers, as the levels of SP‐A and SP‐D correlate with CT image evidence.^36^, ^78^ While CT scans exhibit higher sensitivity compared to chest X‐ray (CXRs) and biomarkers, the utilization of lung radiography and biomarker analysis can prove beneficial in regions lacking access to CT scanning facilities. In addition, some countries are facing restrictions on CT scan devices due to the weight limit of patients with high BMI, which are also considered high risk patients. In such cases, CXRs and biomarkers provide effective alternatives.

Research has shown that both SP‐A and SP‐D are effective defenses against respiratory viruses, but SP‐D appears to be more effective in fighting CoVs. For instance, SP‐D has a higher affinity for binding to the SARS‐CoV‐2 spike protein than SP‐A.^79^

As a predictor, the levels of SP‐D were found to be higher in mild‐moderate pneumonia than in severe/critical infections in COVID‐19.^75^ Additionally, the levels of SP‐D and IL‐6 were significantly elevated in COVID‐19 patients but decreased over time. These biomarkers are closely linked to the development of ARDS and Macrophage Activation Syndrome, Which is a severe condition marked by an intense inflammatory reaction resulting in high fever, altered consciousness, increased susceptibility to bruising, and liver enlargement. It occurs when the innate immune system goes into overdrive, releasing excessive inflammatory substances such as cytokines from macrophages and T‐lymphocytes.^36^ Higher IL‐6 and SP‐D rates were observed in nonsurvivors compared to survivors. In follow‐up studies of COVID‐19 patients, IL‐6, ferritin, CRP, troponin‐I, and D‐dimer were positively correlated with SP‐D levels, while inversely correlated with PaO_2_/FiO_2_ levels.^80^ The elevation observed in pulmonary surfactant levels may be attributed to a compensatory response initiated by the body in the early stages of pneumonia to address the deficiency of surfactant. Nevertheless, as pneumonia advances and inflicts further damage upon alveolar type 2 cells, the concentration of pulmonary surfactant decreases concomitantly with the deteriorating condition of the lungs.^81^ Consequently, the evaluation of fluctuation surfactant levels can serve as a valuable indicator for assessing the progression of COVID‐19 pneumonia, likewise SP‐D has been extensively employed as a biomarker and diagnostic tool for interstitial lung disorders and ARDS.^82^, ^83^ It is worthy to mention that an examination utilizing Cryo‐TEM revealed the capacity of SARS‐CoV‐2 to disrupt the three‐dimensional structure of pulmonary surfactants. The outcomes of this study distinctly indicate that the decline in surfactant levels post‐COVID‐19 infection can be attributed not only to the impairment of type 2 alveolar cells—the producers of surfactants—but also to the direct disruption of the three‐dimensional structure of surfactants. The interplay of these two factors may contribute to an intensified manifestation of respiratory issues associated with COVID‐19.^84^

Surfactant insufficiency, caused by a block in surfactant synthesis in type II alveolar cells, may be responsible for the abrupt worsening of respiratory function in COVID‐19 patients. In the context of managing COVID‐19, the administration of synthetic surfactant to renew or replace depleted endogenous surfactant stores may provide a strong biologic and physiologic rationale for using external surfactant therapy in COVID‐19‐related ARDS.^85^ According to recent research, rhSP‐D has the capacity to prevent SARS‐CoV‐2 cell entrance. This could be due to the protein's function in the innate immune response, which includes protection against inhaled pathogens. In the context of SARS‐CoV‐2, rhSP‐D may inhibit the virus's ability to attach to the ACE2 receptor, inhibiting the virus from entering the cell, this mechanism of action would classify rhSP‐D as a viral entry inhibitor, a category of therapeutics that also includes multivalent antibodies, recombinant ACE2, and peptide.^79^, ^86^

On the other hand, exogenous surfactant has been shown to enhance oxygenation and restore lung function, and hence can be utilized to prevent COVID‐19 pneumonia. It is believed that after meticulous dispersion, exogenous surfactant enters lung alveoli where it comes into contact with the virus. Surfactant molecules bind to spike glycoproteins in the alveolar space and eliminate them.^87^ The surfactant constituent, phosphatidylglycerol, may attenuate COVID‐19‐induced pulmonary pathology through the following mechanisms: First, it may diminish excessive stimulation of the innate immune system by inhibiting TLR‐2 activation induced by microbial components and cellular proteins released from damaged pulmonary cells. This inhibition serves to curtail inflammation and subsequent pulmonary edema. Secondly, there is a potential for phosphatidylglycerol to impede the spread of viral infection to noninfected cells within the pulmonary tissue.^88^ Multiple clinical trials are now being conducted to evaluate the efficacy of surfactant compositions in the treatment of COVID‐19 patients. Variables such as mechanical ventilation technique, treatment schedule, provided dose, administration mode, and the exact formulation used were all considered.^89^

Employing respiratory SPs as biomarkers to assess the severity of COVID‐19 has both advantages and disadvantages. Their direct association with lung function makes them especially intriguing in this context. They have the ability to diagnose diseases earlier and track therapy efficacy more efficiently. Nonetheless, their specificity is fairly limited, and their levels vary significantly between individuals. Furthermore, technical constraints in properly quantifying these proteins provide a challenge, as does their dynamic character over time.

## 4 CONCLUSION

As stated previously, respiratory SPs play a pivotal role in the pathogenesis of COVID‐19. Alterations in hydrophilic surfactant can precipitate acute respiratory distress, while variations in hydrophobic surfactant can serve as valuable indicators for predicting, diagnosing, and monitoring COVID‐19 patients. Investigating pulmonary surfactants, specifically types A and D, and their correlation with COVID‐19 pathogenesis may yield early diagnostic and therapeutic benefits for COVID‐19 infection. The findings underscore the mounting significance of pulmonary SPs in prognostication, detection, and treatment of COVID‐19. However, further research is necessary to comprehensively comprehend these mechanisms and explore their potential therapeutic applications.

## AUTHOR CONTRIBUTIONS

**Mohammad Navid Bastani**: Investigation; writing—original draft. **Shahram Jalilian**: Investigation; writing—review & editing.

## CONFLICT OF INTEREST STATEMENT

The authors declare no conflict of interest.
